# Successful *In Situ* Targeting of Pancreatic Tumors in a Novel Orthotopic Porcine Model Using Histotripsy

**DOI:** 10.1016/j.ultrasmedbio.2023.07.013

**Published:** 2023-08-16

**Authors:** Khan Mohammad Imran, Jessica Gannon, Holly A. Morrison, Juselyn D. Tupik, Benjamin Tintera, Margaret A. Nagai-Singer, Hannah Ivester, Justin Markov Madanick, Alissa Hendricks-Wenger, Kyungjun Uh, David T. Luyimbazi, Michael Edwards, Sheryl Coutermarsh-Ott, Kristin Eden, Christopher Byron, Sherrie Clark-Deener, Kiho Lee, Eli Vlaisavljevich, Irving C. Allen

**Affiliations:** a Graduate Program in Translational Biology, Medicine and Health, Virginia Polytechnic Institute and State University, Roanoke, VA, USA; b Department of Biomedical Sciences and Pathobiology, Virginia−Maryland College of Veterinary Medicine, Blacksburg, VA, USA; c Department of Biomedical Engineering and Mechanics, Virginia Polytechnic Institute and State University, Blacksburg, VA, USA; d Virginia Tech Carilion School of Medicine, Roanoke, VA, USA; e DeBusk College of Osteopathic Medicine, Lincoln Memorial University, Knoxville, TN, USA; f Division of Animal Science, College of Agriculture Food and Natural Resources, University of Missouri, Columbia, MO, USA; g Department of Surgery, Carilion Clinic and Virginia Tech Carilion School of Medicine, Roanoke, VA, USA; h Department of Small Animal Clinical Sciences, Virginia−Maryland College of Veterinary Medicine, Blacksburg, VA, USA; i Department of Large Animal Clinical Sciences, Virginia−Maryland College of Veterinary Medicine, Blacksburg, VA, USA

**Keywords:** Xenograft, Pancreatic cancer, Biomedical device, Pre-clinical animal model, Focused ultrasound, Tumor ablation

## Abstract

**Objective:**

New therapeutic strategies and paradigms are direly needed to treat pancreatic cancer. The absence of a suitable pre-clinical animal model of pancreatic cancer is a major limitation to biomedical device and therapeutic development. Traditionally, pigs have proven to be ideal models, especially in the context of designing human-sized instruments, perfecting surgical techniques and optimizing clinical procedures for use in humans. However, pig studies have typically focused on healthy tissue assessments and are limited to general safety evaluations because of the inability to effectively model human tumors.

**Methods:**

Here, we establish an orthotopic porcine model of human pancreatic cancer using *RAG2/IL2RG* double-knockout immunocompromised pigs and treat the tumors *ex vivo* and *in vivo* with histotripsy.

**Results:**

Using these animals, we describe the successful engraftment of Panc-1 human pancreatic cancer cell line tumors and characterize their development. To illustrate the utility of these animals for therapeutic development, we determine for the first time, the successful targeting of *in situ* pancreatic tumors using histotripsy. Treatment with histotripsy resulted in partial ablation *in vivo* and reduction in collagen content in both *in vivo* tumor in pig pancreas and *ex vivo* patient tumor.

**Conclusion:**

This study presents a first step toward establishing histotripsy as a non-invasive treatment method for pancreatic cancer and exposes some of the challenges of ultrasound guidance for histotripsy ablation in the pancreas. Simultaneously, we introduce a highly robust model of pancreatic cancer in a large mammal model that could be used to evaluate a variety biomedical devices and therapeutic strategies.

## Introduction

Histotripsy has recently been suggested as an alternative tumor ablation modality that could prove useful in treating pancreatic cancer [1]. Histotripsy is a non-ionizing, non-thermal and image-guided non-invasive tissue ablation technique capable of treating solid tumors [2]. Histotripsy using high pressure (>10 MPa) and very short duration (<20 μs) generates a distinctive cavitation “bubble cloud” at the focus of which expansion and collapse create high stress to completely disintegrate target tissue into an acellular homogenate with no residual cellular structures [3−8]. As a non-thermal modality, histotripsy has been successfully used to ablate tumors and healthy tissues near vital organs, such as the spleen, liver, major vessels, nerves, and bile ducts without causing damage [1,4,9−12]. Histotripsy has also been used in the highly vascular liver to generate full ablation successfully and consistently [13,14]. This includes the completion of the first-in-human use of histotripsy to treat hepatic tumors, reported earlier in 2022 [15]. This complements several pre-clinical rodent studies and veterinary clinical trials that have reported successful ablation of osteosarcoma, cholangiocarcinoma, pancreatic tumors and spontaneous soft tissue [1,9,10,16−19].

For all of the tumor ablation modalities, including histotripsy, key data are required for the establishment of ablation parameters for each targeted tissue. *Ex vivo* tumor tissue and healthy organs typically lack accurate mechanical properties and *postmortem* tissues usually become unsuitable for ablation modality testing [20,21]. Murine models have certain advantages that make them appealing, such as ease of housing and handling, cost, genetic similarities to humans and availability of numerous established lines of interest [22−24]. However, the significant discrepancy in size, anatomy and genetic makeup between mice and humans and ablation volume are major challenges when translating ablation findings from mice to humans. Veterinary clinical trials can also be conducted to evaluate treatment effectiveness on spontaneous tumors. However, these studies can often lack statistical power, the ability to conduct robust and highly controlled experimental studies can be limited and reagent availability for mechanistic studies can be a limitation.

Pigs have proven to be a useful model for biomedical device development and tumor ablation trials, especially in the context of designing human-sized instruments, improving surgical techniques and optimizing clinical procedures for use in humans [4,9,20,25−27]. However, to date, pig studies have focused on healthy tissue assessments and are limited to general safety evaluations. Although critical for translation to human patients, the focus on healthy tissues is a significant limitation. As mentioned above, healthy tissue studies do not account for physical, mechanical, physiological and biological differences that exist between healthy tissue and tumor. Thus, to circumvent these limitations, we generated unique *RAG2/IL2RG*-deficient immunocompromised pigs [28] and previously determined their ability to be engrafted subcutaneously with human xenograft tumor cell lines [20]. Here, we extend these prior subcutaneous studies and conduct orthotopic engraftment of human Panc-1 cells in the pig pancreas. We demonstrate the utility of this model in accurately and consistently generating tumors in specific locations of the pancreas and further characterize the tumors generated with those derived from comparable mouse pancreatic tumors (Pan02) and specimens from human patients. To further illustrate the utility of this model in ablation therapy development, we successfully targeted a subset of the pancreatic tumors with histotripsy and determined for the first time the feasibility of using histotripsy to treat pancreatic cancer. This work serves as an effective proof-of-concept study and has identified both strengths and challenges in targeting pancreatic tumors.

## Methods

### Generation of immunocompromised pigs by RAG2/IL2RG deletion

*RAG2/IL2RG* double-knockout pigs were generated using CRISPR/Cas9 as previously described (Figs. S1 and S2, online only) [28−30]. A sterile hysterectomy method was used to deliver piglets from the sow that were immediately aseptically transferred to isolators for housing under germ-free conditions [31]. All study methods were in compliance with the National Institutes of Health (NIH) *Guide for the Care and Use of Laboratory Animals* and with the Virginia Tech Institutional Animal Care and Use Committee (IACUC). The immunocompromised status of each pig was validated through genotyping and sequencing to confirm *RAG2/IL2RG* double-knockout status (Fig. S3, online only).

### Ethics

All human specimens were collected and used according to institutional review board (IRB) guidelines following institutional approval. All animal experiments were approved and carried out in accordance with the Virginia Tech Institutional Animal Care and Use Committee under IACUC Protocol 19–196-CVM, approved December 15, 2021, and 22–047-CVM, approved March 31, 2022.

### Surgery and implantation of Panc-1 human pancreatic cancer cells into pig pancreas

Human pancreatic ductal epithelial carcinoma Panc-1 cells (ATCC, Manassas, VA, USA) were grown in Dulbecco’s modified Eagle’s medium (DMEM) supplemented with 10% fetal bovine serum and 1% Normocin. To generate a tumor with a defined margin, cells were resuspended in Matrigel at a concentration of 6 × 10^6^/100 *μ*L and kept on ice. One-week-old pigs were individually placed under anesthesia for surgery and injected in the pancreas with 100 *μ*L of Panc-1 cells; tumors were permitted to grow for about a month (25−30 d) before histotripsy (Figs. S1, S2B and S2C; Table 1). In the present study, the pigs were treated as follows: 1 pig was used as a control (Table 1, no surgery or tumor injection); 6 pigs were used to validate the surgery, characterize orthotopic tumor progression and identify optimal acoustic windows for ultrasound visualization and histotripsy treatment (Table 1); 3 pigs were used to conduct proof-of-concept studies using histotripsy (Table 1).

### Ex vivo *histotripsy treatment*

Histotripsy was tested in the excised Panc-1 tumors from the *RAG2/IL2RG* pigs *ex vivo* using the same transducer. Tumors were de-gassed, embedded in gelatin phantoms and treated as previously described [6,20]. Histotripsy was applied to the first and second tumor samples at ~14 MPa peak negative pressure with an ~1.5 cm diameter spherical ablation volume. In addition to the excised tumor samples from pigs treated with histotripsy, a human excised pancreatic tumor sample (stage IIA, freshly received in 0.9% de-gassed saline) was also treated and compared with the Panc-1 tumors.

### In vivo *histotripsy treatment*

One week prior to treatment, pigs were fed a diet that included sweetened custard with simethicone and bisacodyl to minimize the intestinal contents and gas [32,33]. All animals were sedated using 2−4 mL/kg of Telazol−Ketamine−Xylazine (TKX) and kept under anesthesia throughout all procedures. The pigs’ weight range at the time of treatment was 6−7 kg. Histotripsy was performed by targeting each orthotopic Panc-1 tumor *in vivo/in situ* using a custom 32-element 500 kHz therapy transducer driven by a custom high-voltage field programmable gate array (FPGA) board (Altera DE0-Nano Terasic Technology) and pulser to deliver short histotripsy pulses <2 cycles. The therapy transducer has a focal depth of 78 mm with transverse and elevational aperture dimensions of 128 and 112 mm, respectively. Further device specifications are previously reported [1]. A robotic micropositioner connected the transducer to a histotripsy treatment cart (HistoSonics) with a 3 MHz curvilinear imaging probe (Model C52, Analogic Corp., Peabody, MA, USA) coaxially aligned with the therapy transducer to allow for real-time imaging feedback for treatment monitoring as detailed previously [1]. To target the pancreatic tumors, a veterinary radiologist (M.E.) used free-hand ultrasound imaging (Model C5–2, Analogic Corp.) to identify the tumors and mark the best acoustic window for histotripsy on the abdomen of the pig (Fig. S2D). Single-cycle histotripsy pulses at a pulse repetition frequency (PRF) of 500 Hz were applied through an automated volumetric spherical ablation strategy that moved through the tumor volume, twice delivering a total of ~1000 pulses per treatment point (PPP). To allow overlap of the bubble cloud at each treatment point, points were spaced by 3.5 mm in the axial direction and 1.5 mm in the lateral and elevational directions. Pressure was slowly increased until a bubble cloud was generated in the targeted tumor. The *in situ* focal pressures could not be directly measured non-invasively. With 3−4 cm of overlying tissue and using 0.5 dB/cm-MHz attenuation for the overlying tissue, the in situ peak negative pressure was estimated to be ~31.2 MPa. However, it is worth noting that this is likely an overestimate of the actual *in situ* pressure as this estimate does not include pressure loss caused by gas blockage and tissue aberration, which recent work has indicated can be significant for intra-abdominal histotripsy procedures [34]. Tumors were treated twice with single-cycle histotripsy pulses at a PRF of 500 Hz. A full necropsy was performed immediately after the treatment by a veterinary pathologist (S.C.O./K.E.).

### Histopathology

All tissues were fixed in formalin and stained with hematoxylin and eosin (H&E) or trichrome. Histology sections were prepared at 5 mm thickness with 20 or 50 mm intervals from multiple sections of tumor to find start, middle and end of the ablation zone based on the assessment of a board-certified pathologist. To verify the tissue ablation, regions of cellular damage within and outside the ablation zone were assessed. Trichrome-stained images were scored using Fiji. Images were subjected to color deconvolution into trichrome using Fiji, and only blue-stained collagen sections were analyzed, as previously described [35].

### Blood chemistry

Blood was collected from animals to evaluate pancreatitis. Blood samples were collected from the jugular vein using un-supplemented BD Vacutainer blood collection tubes, and serum was separated following centrifugation to remove the red blood cells. Levels of amylase and lipase were evaluated in the serum.

## Results

### Successful generation of a pig orthotopic pancreatic tumor model using the Panc-1 human pancreatic cancer cell line

We generated 10 *RAG2/IL2RG* pigs using the CRISPR/Cas9 system as previously described [9,28,29]. Briefly, we used 1265 *in vitro* fertilized oocytes where the porcine *RAG2* and *IL2RG* genes were targeted using CRISPR/Cas9 prior to *in vitro* culture. Four days after *in vitro* fertilization (IVF), 179 developing embryos between the eight-cell and morula stages were transferred to surrogate gilts. Genomic DNA collected from ear notches revealed that RAG2 and IL2RG were successfully targeted in all 10 piglets (Fig. S3). Our engraftment rate for the orthotopic Panc-1 cell line in the *RAG2/IL2RG* immunocompromised pigs is 90.9%. Metastatic tumors were noted in one animal (Table 1; Fig. S4). Of note, none of the pigs developed pancreatitis, based on normal blood ranges of amylase and lipase (Fig. S5, online only). Our results illustrate our ability to successfully engraft orthotopic human Panc-1 tumors in *RAG2/IL2RG* knockout pigs.

### Ex vivo *excised tissue used to define histotripsy parameters for pancreatic tumor ablation*

**Figure 1.**
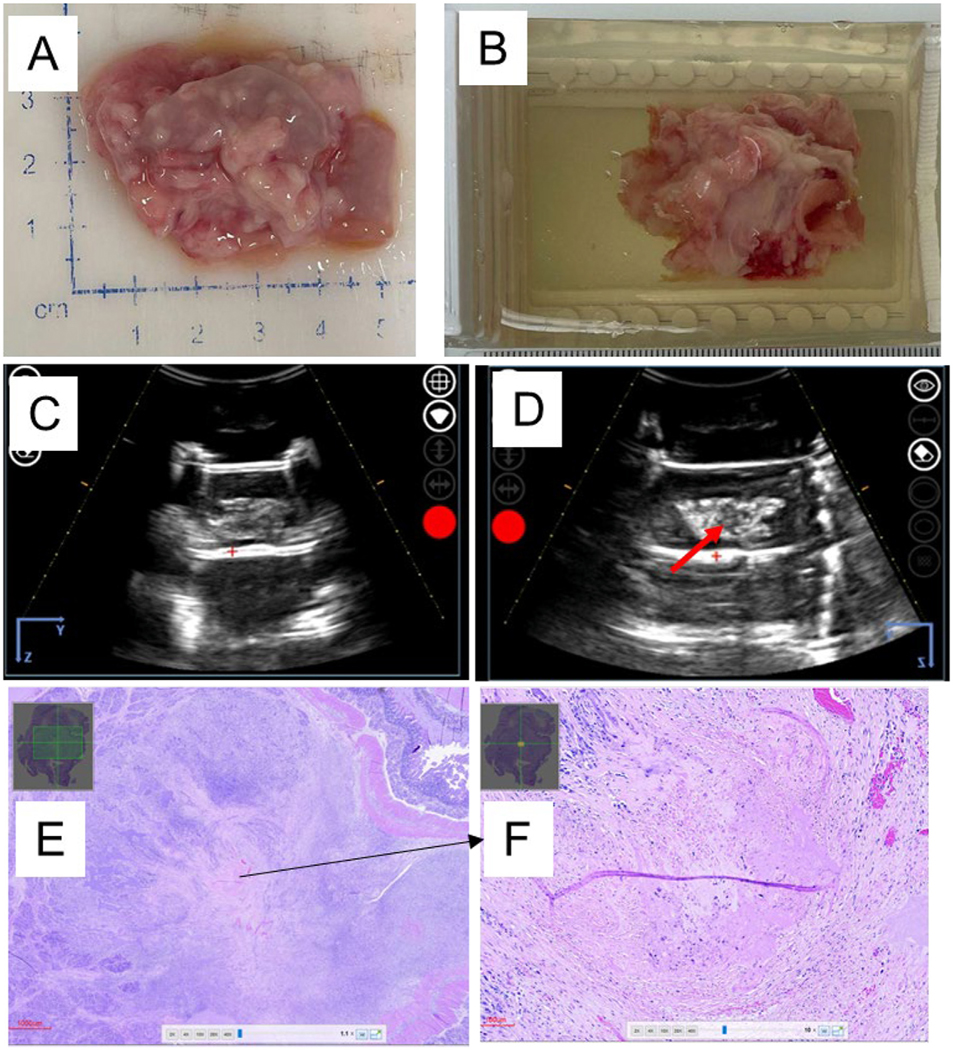
Histotripsy optimization using *ex vivo* tumor treatment. (A) Gross image of the excised Panc-1 tumor. (B) Excised Panc-1 tumor inside Matrigel block. (C, D) Ultrasound image of the Panc-1 tumor inside the block (C) before and (D) after treatment (*red arrow* points to hypo-echoic region after treatment). (E, F) Histopathology assessments were used to characterize the ablation zone after hematoxylin and eosin staining using images at (E) 1.1 ×, bar = 1000 μm and (F) 10 ×, bar = 100 μm. Ablated cells stain more pink in hematoxylin and eosin than healthy cells.

**Figure 2.**
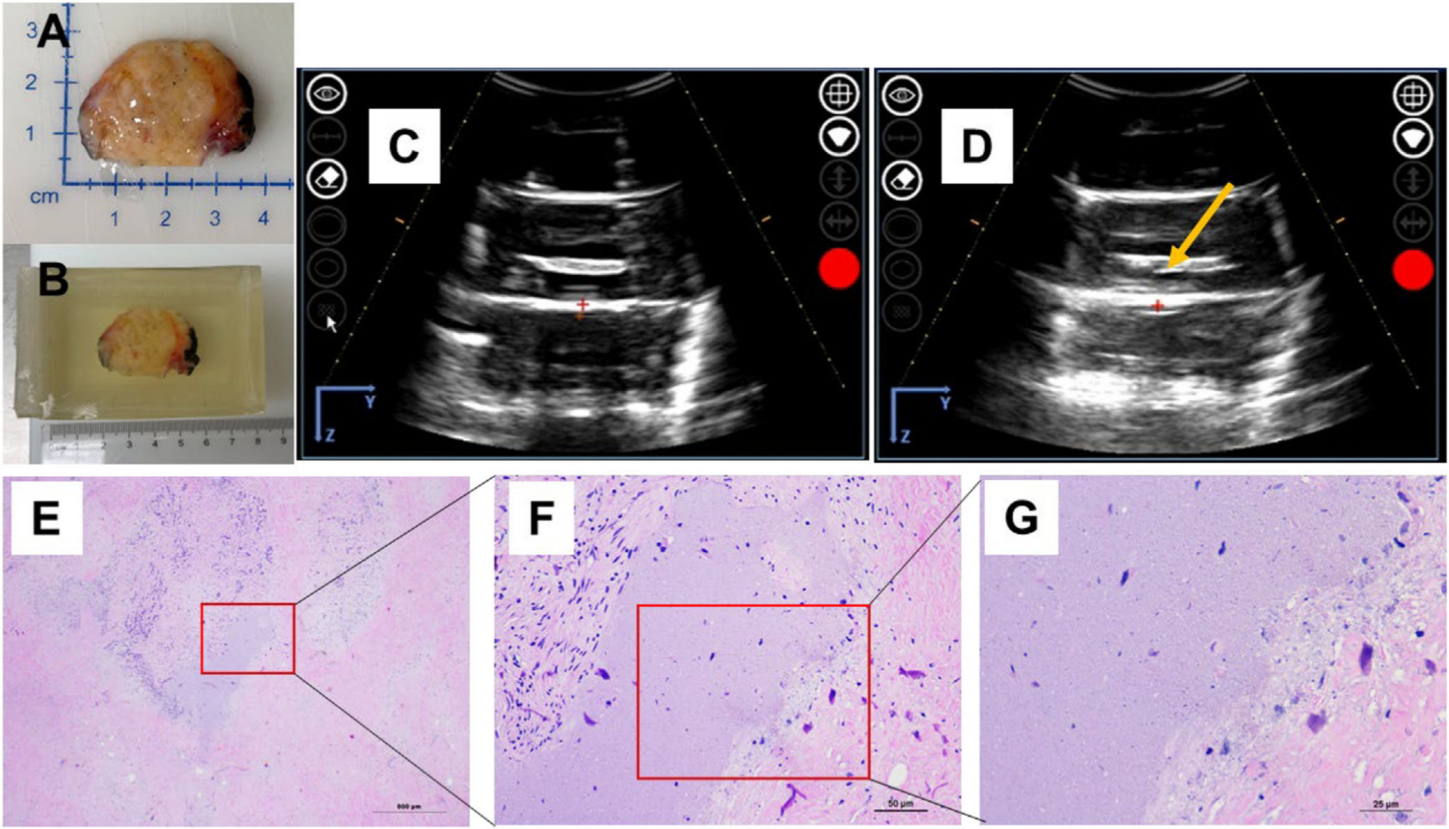
Human pancreatic tumors have an ablation profile similar to that of the pig-derived Panc-1 tumor. (A) Gross image of the excised human pancreatic tumor. (B) Excised tumor embedded in Matrigel block. (C, D) Ultrasound images of the pancreatic tumor inside the block (C) before and (D) after treatment (*yellow arrow* points to hypo-echoic region after treatment). (E−G) Histopathology assessments were used to characterize the ablation zone after hematoxylin and eosin staining using images at (E) 4 ×, (F) 20 × and (F) 40 ×, with scale bars of 500, 50 and 25 μm. respectively. Insets focus on the ablation zone.

To refine and optimize the histotripsy parameters for *in vivo* studies, orthotopic tumors were excised from pigs and human patients (IRB No. 20–949). Tumors excised from mice were ~1 cm in diameter (not shown), orthotopic pig tumors were ~3 cm in diameter and the human tumor was ~3 cm in diameter (Figs. 1A and 2A). The excised pig tumors were pathologically characterized as corresponding to stage IIA human pancreas ductal adenocarcinomas. The histotripsy parameters were gradually increased until a bubble cloud was identified in the targeted region of the tumor on ultrasound imaging. This was noted at ~13.6−14.1 MPa peak negative pressure, which was applied to an estimated 1, 1.4 and 1.5 cm diameter spherical ablation volumes in the human and two excised pig tumors, respectively (Figs. 1C, 1D, 2C and 2D). Histopathology revealed that tumor tissue was successfully ablated in tumors from both pig and human patients under these conditions (Figs. 1E, 1F and 2E–G). Ablations were confirmed to be partial, with the remaining Panc-1 cells generally dispersed in small pockets throughout the ablation zone. Ablated tissues were characterized by loss of recognizable tissue architecture and replacement by fine basophilic (bluish) debris. Larger pockets of cells were visible along the margins, with a clear demarcation between the ablation zone and untreated tumor (Figs. 1E, 1F and 2E–G). This is consistent with previously reported findings from the use of histotripsy to treat *ex vivo* mouse Pan02 subcutaneous tumors with partial ablation [18].

### *Histotripsy successfully ablates orthotopic pancreatic tumors* in situ

**Figure 3.**
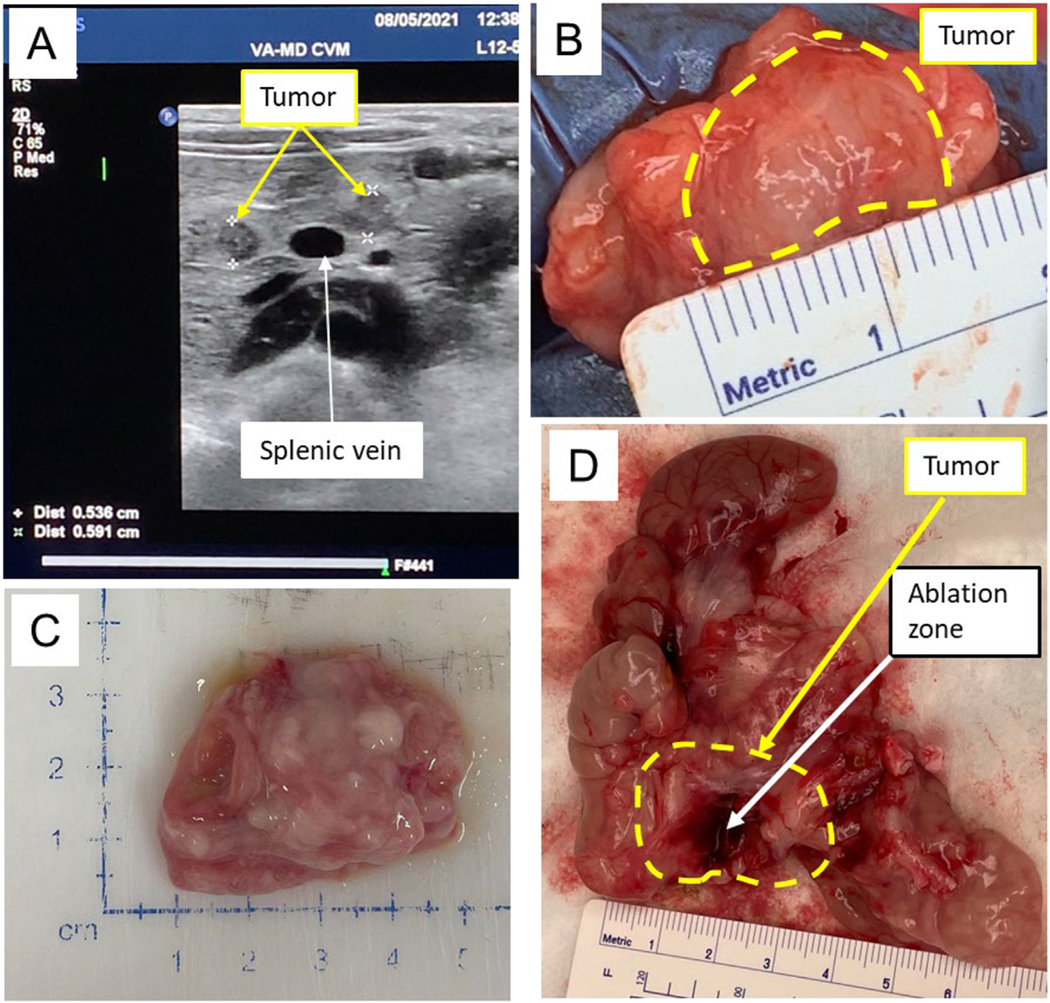
Histotripsy successfully ablated orthotopic Panc-1 tumors in the pig pancreas under clinically relevant conditions. (A) Orthotopic Panc-1 tumors were readily identifiable using ultrasound and were engrafted in areas adjacent to critical structures (such as the splenic vein, *white arrow*) or in areas visually devoid of critical structures to allow for limitation testing. Tumors are marked with the white “+” and *yellow arrows* which are more hypoechoic region in B-mode ultrasound. (B, C) Tumors were verified and characterized after necropsy, both (B) in the pancreas (*yellow dashed line*) and (C) after excision. (D) Histotripsy ablation zones were readily identified in gross specimens after treatment, with the tumor outlined by the *yellow dashed line* and *yellow arrow* and the ablation zone indicated by the *white arrow*. The ablation zone appears darker from the hemorrhage and edema caused by the mechanical force of the bubble cloud.

For all pigs, prior to treatment, pancreatic tumors were visualized using ultrasound imaging using a freehand ultrasound imaging probe with pressure applied to the abdomen to displace bowel gas (Fig. 3A). Although some tumors were readily identifiable by ultrasound and effectively targeted with histotripsy, some of the pancreatic tumors could not be consistently visualized by the co-axially aligned imaging probe because of the bowel gas and the inability to apply significant pressure. Under these conditions, the focus of the histotripsy transducer was aligned to pre-marked anatomical landmarks. Histotripsy was applied at approximately >31.2 MPa peak negative pressure. These *in vivo* pressure levels were notably higher than the *ex vivo* histotripsy ablations, which used 14 MPa peak negative pressure. The difference in pressures can likely be attributed to aberration and gassy effects in the overlying tissues exhibited *in vivo* resulting in the higher estimated pressures reported for generating the bubble clouds *in vivo*.

Histotripsy was applied through an automated volumetric ablation strategy that moved through the tumor volume twice with a total PPP for the complete treatment of ~1000. The first and second pigs were treated with a 1.75 and 1.90 cm diameter spherical ablation volumes, respectively. With tumors ~2 cm in diameter in each pig, these treatments corresponded to ~67% and ~86% of the tumor volumes targeted with histotripsy, respectively. During treatment, histotripsy cavitation was audible, but the bubble cloud was not clearly identifiable on ultrasound imaging in all tumors.

**Figure 4.**
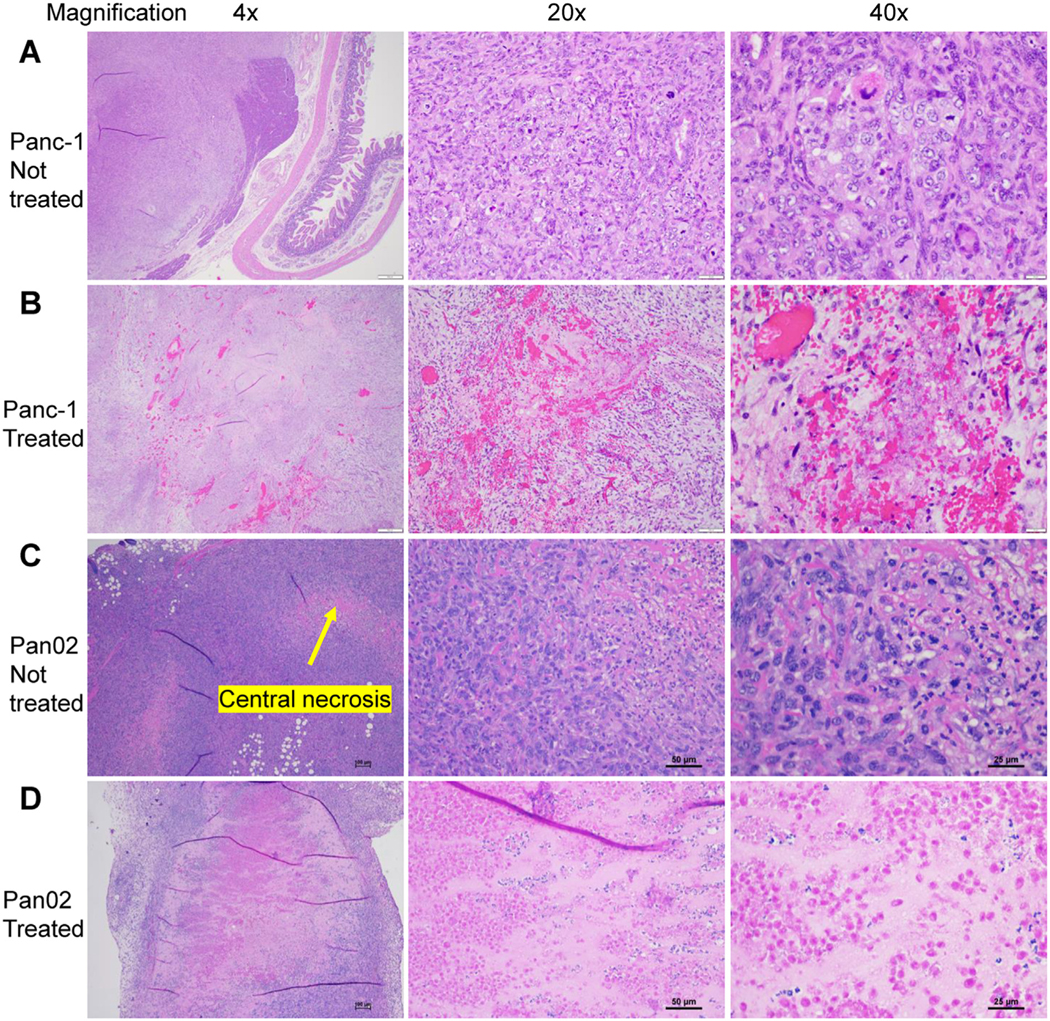
Histopathology characterization of treated and untreated Panc-1 tumors with mouse Pan02 tumors. (A, B) Human Panc-1 tumors after either (A) no treatment or (B) histotripsy treatment and evaluation by histopathology using hematoxylin and eosin−stained sections. Histotripsy treatment resulted in dispersed ablation zones within tumor. (C) Panc-1 tumors exhibited significantly different histopathological features compared with subcutaneously engrafted mouse Pan02 tumors engrafted in immunocompetent C57Bl//6 mice. Subcutaneously engrafted mouse Pan02 tumors exhibit central necrosis (indicated by *yellow arrow*) not found in human Panc-1 tumor grown in pig pancreas. (D) Histotripsy was also highly effective in ablating the Pan02 tumors in mice, resulting in clear ablation margins with decellularization. From left to right, magnifications are 4 ×, 20 × and 40 ×, and scale bars are 500, 50 and 25 μm, respectively.

After treatment and necropsy, tumor characteristics were noted, and both targeted and non-targeted ablation zones were evaluated (Fig. 3B–D). Gross morphology of the treated pancreatic tumors revealed a well-defined histotripsy ablation zone, which represented a partial tumor ablation (localized hemorrhage) (Fig. 3D). Ablation zones were approximately 1.0 cm in size (Fig. 3D). No off-target damage to surrounding organs was noted. A successful but partial ablation in the pancreatic tumor was verified by histopathology evaluation (Fig. 4A, 4B). Despite issues with gas in the gastrointestinal tract and the lack of robust image guidance, these studies indicate for the first time that histotripsy, even under suboptimal conditions, can ablate pancreatic tumors *in vivo/in situ* under clinically relevant conditions.

### Orthotopic Panc-1 tumors in pigs share similar pathological characteristics with other common cell line−based pancreatic tumors before and after histotripsy treatment

The majority of studies evaluating therapeutic approaches targeting pancreatic cancer use rodent models, such as the mouse Pan02 model [18,36−38]. Thus, we next sought to compare the human Panc-1 tumors grown in the immunocompromised pigs with the murine Pan02 tumors propagated in wild-type mice [9,18,20]. In general, the Panc-1 morphological characteristics of tumors from both immunocompromised pigs and Pan02 tumors from immunocompetent mice were highly similar (Fig. 4A–D). Both tumors were both densely cellular, although we observed areas of central necrosis of irregular size within the Pan02 murine tumors that were not observed in the Panc-1 tumors (Fig. 4C). In both tumor types, spindle-shaped cells were irregularly arranged without any distinct pattern. For histotripsy treatment, we attempted to use a partial treatment parameter that was not designed to treat the whole tumor (Fig. 4B, 4D). Here, we report that subcutaneous murine Pan02 tumor targeting and ablation (Fig. 4D) have improved efficacy compared with the orthotopic Panc-1 tumor (Fig. 4B) based on the generally welldefined and larger ablation zones in treated murine tumors.

**Figure 5.**
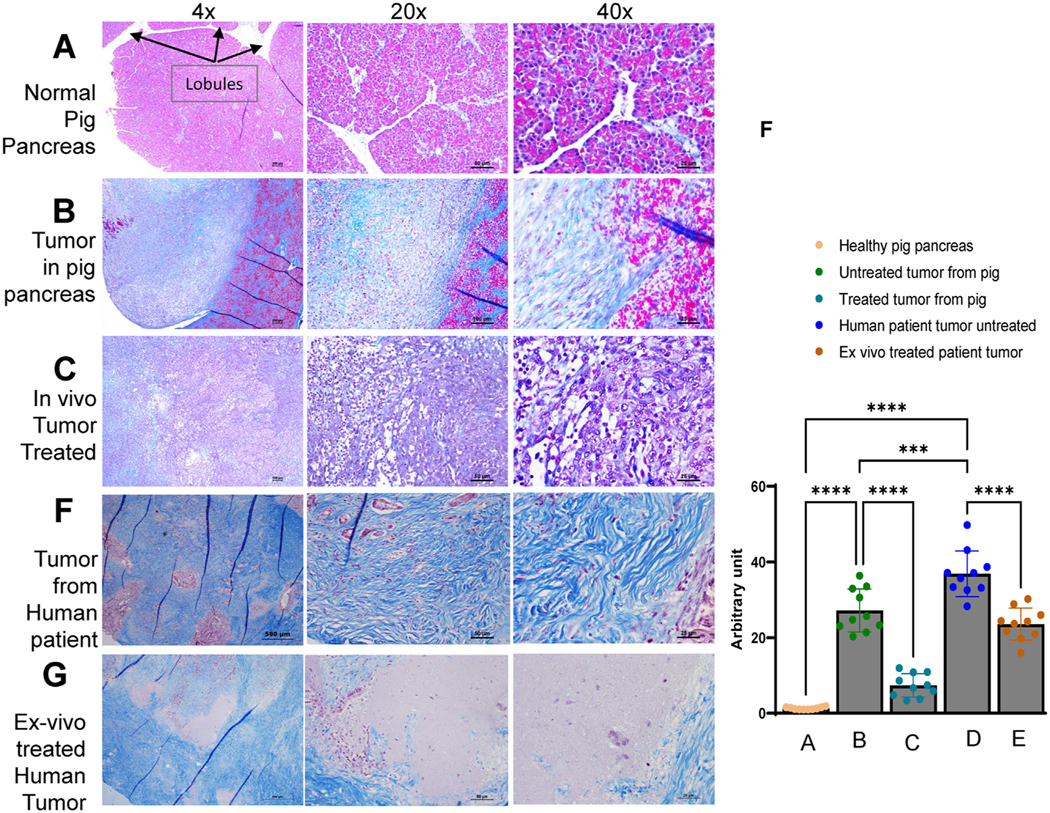
Histotripsy significantly reduced the stromal compartment and collagen levels in the orthotopic Panc-1 tumors in the pig pancreas *in situ* and also *ex vivo* treated human patient tumor. (A−C) Healthy and tumor specimens were stained with trichrome for histopathology assessments. (A) Trichrome assessments of the healthy pig pancreas revealed very minimal collagen content (*arrows* indicate lobules of healthy pancreas). (B) The orthotopic Panc-1 tumors exhibited dense collagen staining (blue). (C) Histotripsy significantly reduced the collagen levels and generated large tracks of collagen and stroma-free areas in the tumor tissue. (D) Patient tumor exhibits very high content of collagen. (E) *Ex vivo* partially treated patient tumor exhibits reduced collagen content in the treated areas. From left to right, magnifications are 4 ×, 20 × and 40 ×, and bars are 500, 50 and 25 μm, respectively. (F) Fifteen images from groups A−E were scored for *blue* (collagen) staining using FIJI. Scoring indicates reduced collagen after histotripsy treatment (bar B vs. bar C and bar D vs. bar E). ****p < 0.00001, ***p < 0.0001.

Previous studies have established the micro-heterogeneity of collagen in human pancreatic tumor types with the collagen density associated with the tumor biomechanical stiffness and inversely related to vascular perfusion [39,40]. The Panc-1 tumors in the pig pancreas were stiffer than the normal pancreas measured with B-mode ultrasound (Fig. 3A) and were readily identifiable grossly from the surrounding pancreas tissue during necropsy. B-mode (brightness mode) images reveal the relative stiffness of lesions compared with the stiffness of surrounding softer tissue. Stiffer areas deform less easily than their surroundings and are depicted in darker (hypo-echoic) images, whereas softer areas deform more easily than their surroundings and are depicted as lighter areas in the images. Malignant masses typically appear dark and have high contrast against the background in pancreas. The hypoechoic region is clearly visible in Figure 3A (*yellow arrow*, within stars) and the masses were readily identified by touch during the necropsy. The healthy pig pancreas is clearly divided into multiple lobules by connective tissue septae (Fig. 5A). Tumor tissue was characterized by a loss of normal lobular architecture and replacement by tumor cells embedded in an abundant collagenous stroma (*blue*) (Fig. 5B). After histotripsy treatment, significantly less collagen was observed (B vs. C in Fig. 5F graph). Of note, the untreated pancreatic tumor from the human patient had significantly higher collagen than the untreated tumor grown in pig pancreas (Fig. 5B, 5D, 5F). However, *ex vivo* histotripsy treatment of the tumor tissue from the human patient exhibited reduced collagen when compared with before treatment (Fig. 5B, 5D, 5F). Taken together, our data suggest that the orthotopic pig model depicts nodular tumor formation like the mouse model and better reflects the collagenous stroma formation seen in the human patient tumor. Data also suggest that histotripsy is able to effectively ablate tumor tissue, along with collagenous materials and stroma.

## Discussion

Histotripsy is a promising ablation modality because of its non-thermal, non-ionizing and non-invasive nature. Nevertheless, along with other highly promising ablation modalities, clinical translation of histotripsy to treat pancreatic cancer has been hindered by the lack of vigorous and clinically relevant pre-clinical animal models. In this study, we establish an orthotopic and highly reproducible human pancreatic tumor model in swine that accurately and effectively mimics many of the clinical challenges expected to be faced in human patients. The ability to surgically engraft tumors within the pancreas in specific areas that are difficult to treat and/or in areas adjacent to critical anatomical structures in the pancreas allows for highly robust limitation, proof-of-concept and proof-of-principle testing.

Using this unique model, we report for the first time the successful histotripsy ablation of pancreatic tumors *in situ* under clinically relevant conditions. Histotripsy successfully destroyed tumor tissue in the ablation zone with no adverse effects observed during the monitoring period. Within the ablation zone, individual and small pockets of Panc-1 cells were still observed, mostly at the margins of the treatment zones and in areas outside of the targeted region of the organ. Gas in the overlaying gastrointestinal tract was a significant limitation. The lack of a clear bubble cloud in some of the targeted tumors, in part because of gas, may necessitate more invasive approaches to remove gas in difficult-to-visualize targets.

Although we were successful in creating ablations and significantly debulking the tumors, we did not achieve a full ablation in any of the targets. Similar to human patients, the Panc-1 tumors generated in this study exhibited a dense stromal region that has altered the mechanical properties of the tumors and appeared to reduce histotripsy effectiveness. This is typical of pancreatic tumors, which can be highly heterogenous in terms of anatomy, cellular constituents and stromal density [41,42]. Thus, each of these characteristics should be taken into account for not only histotripsy, but any local tumor ablation modality. These data will be used to inform, optimize and refine our approaches using these unique animal models in future studies.

There is currently extensive debate in the tumor ablation field regarding the benefits of partial ablation versus full ablation and the order of operations in terms of treatment timing and surgical intervention [43,44]. Much of this stems from the desire to optimize the host immune response following treatment. Local tumor ablation can augment immune system activation to promote the clearance of the remaining residual cancer cells in the treatment zone and at the margins following ablation [45−52]. Local tumor ablation can also promote the systemic anti-tumor adaptive immune response to target the micro-metastatic lesions in the primary organ and the metastatic tumors at distal sites [45−50]. Thus, the benefits of ablation modalities such as histotripsy have exciting potential to extend beyond the local ablation zone, but more work is necessary to better define the underlying biology of immune system activation to inform the ablation modality treatment parameters. However, what is clear from the data presented here is that even partial histotripsy ablation is capable of significantly reducing the stromal niche, specifically the collagen composition, of the Panc-1 tumors (Fig. 5F). This suggests that histotripsy may be effective in increasing therapeutic access to the targeted tumor. The dense stroma is a significant limitation for many of the current frontline therapeutics for pancreatic cancer [53−55]. Thus, by degrading this stromal compartment, histotripsy may be an effective co-therapeutic approach for many of these current and emerging therapeutic approaches that previously had limited effectiveness.

The use of local tumor ablation modalities, such as histotripsy, to induce a robust and reproducible systemic, anti-tumor immune response to effectively eliminate cancer cells in the untreated margins and at metastatic sites is an emerging area of research interest [18]. Unfortunately, while the lack of an intact immune system enables human tumor cell line and PDX engraftment in the *RAG2/IL2RG* pigs described here, this model does not effectively allow assessments of the immune system niche. Thus, as we report here, although this model is ideal for reproducing mechanical and physical tumor properties, it is limited in terms of evaluating the use of co-therapeutic approaches based on immune system augmentation. For example, prior work has had promising results in mouse models combining histotripsy approaches with immune checkpoint inhibitors [56,57]. These studies reveal improved immune checkpoint blockade therapy of poorly immunogenic pancreatic tumors. To better evaluate the pancreatic cancer immune niche in pigs, recent work has resulted in the development of a porcine pancreatic cell line that can be used in immunocompetent animals [58,59]. However, the model still requires optimization before it can be fully deployed [58,59]. Limitations of this immunocompetent model currently include a lack of investigation of the tumor microenvironment and stroma formation compared with human pancreatic tumors [58,59]. To circumvent these issues, the use of transgenic pigs with Cre-inducible *TP53^R167H^* and *KRAS^G12D^* mutations has also been developed to model pancreatic cancer in immunocompetent animals [60]. The tumors generated in these “Oncopigs” are undifferentiated carcinomas with a significant inflammatory component similar to that of pacreatobiliary carcinomas in human patients [60]. However, there are several limitations to the Oncopig model [58,60,61]: surgery is necessary to place a gelatin sponge necessary for the delivery of the adenoviral vector for Cre induction, the tumors themselves appear highly inflammatory as opposed to the generally immuno-suppressive tumor microenvironment commonly observed in human patients, the tumors are poorly differentiated and hypovascular, cost and scale are common concerns with this model, the reliance on the adenovirus vector can create tumors that are difficult to predict and localize and tumors can take over a year to reach <1 cm in size [58,60,61]. It should be noted that in our current study, the size of the pig could be considered a limitation. Here, we use piglets rather than adult pigs. The immunocompromised status of the piglets allows us to grow human PDAC tumors; however, their immunocompromised status requires that they be housed in isolators that place limits on animal size. The animals used in the current studies were allowed to grow to the maximum size possible for the isolators used. Based on the successful results of the current study, future studies will transfer animals to larger isolators to allow for the animals to reach more clinically relevant sizes. Thus, although the pancreatic cancer pig model characterized in the current work has limitations, this model also has several strengths over the previously described models that should be considered for studies moving forward.

## Supplementary Material

MMC1

## Figures and Tables

**Table 1:** General characteristics of tumor growth

Pig ID	No. of injections/tumor found	Size of tumor (cm)	Location in pancreas	Local or systemic	Duodenum infiltration	Model use

111	3/3	2.5 × 0.5 head, 1 × 0.7 head, 2.1 × 1.2 tail	Head, head, tail	Local	Yes	Surgery validation, side effects monitoring, tumor growth monitoring, acoustic window identification
112	0	NA	NA	NA	NA	
113	2/2	1.6 × 0.8 head, 2.0 × 1.2 tail	Head, tail	Local	No	
115	2/2	1.3 × 1.1 head, 1.5 × 0.9 tail	Head, tail	Local	No	
116	3/3	1.5 × 0.7 head, 1.0 × 0.7 head, 0.9 × 0.6 tail	Head, head, tail	Local	Yes	
117	3/2	1 × 1 head, 2 × 1 tail	Head, body, tail	Local	No	
118	3/3	1.6 × 1 head, 1 × 0.9 head, 1 × 1 tail	Head, body, tail	Local	No	
121	2/1	1 × 1 head	Head	Local	No	Feasibility of histotripsy treatment
122	2/2	1.6 × 1.9 head (2 tumors merged)	Head	Local	No	
123	2/TNTC	2.1 × 1.4 head, 0.9 × 0.8 tail (primary tumors)	Head and tail of pancreas,* other organs (stomach, intestine, spleen)	Systemic	Yes	

Engraftment success rate is 20/22 = 90.9%.

TNTC, too numerous to count.

* Number, size and location of human Panc-1 tumors grown in pig pancreas. Head, body and tail indicate locations in the pig pancreas where the tumors were engrafted and localized.

## Data Availability

The data that support the findings of this study are available on reasonable request from the corresponding author.

## References

[1] Hendricks-Wenger A, Arnold L, Gannon J, Simon A, Singh N, Sheppard H, Histotripsy ablation in preclinical animal models of cancer and spontaneous tumors in veterinary patients: a review. IEEE Trans Ultrason Ferroelectr Freq Control 2021;69:5–26. 34478363 10.1109/TUFFC.2021.3110083 PMC9284566

[2] Xu Z, Hall TL, Vlaisavljevich E, Lee FT Jr. Histotripsy: the first noninvasive, non-ionizing, non-thermal ablation technique based on ultrasound. Int J Hyperthermia 2021;38:561–75. 33827375 10.1080/02656736.2021.1905189 PMC9404673

[3] Maxwell AD, Cain CA, Hall TL, Fowlkes JB, Xu Z. Probability of cavitation for single ultrasound pulses applied to tissues and tissue-mimicking materials. Ultrasound Med Biol 2013;39:449–65. 23380152 10.1016/j.ultrasmedbio.2012.09.004 PMC3570716

[4] Vlaisavljevich E, Kim Y, Allen S, Owens G, Pelletier S, Cain C, Image-guided non-invasive ultrasound liver ablation using histotripsy: feasibility study in an in vivo porcine model. Ultrasound Med Biol 2013;39:1398–409. 23683406 10.1016/j.ultrasmedbio.2013.02.005 PMC3709011

[5] Vlaisavljevich E, Maxwell A, Mancia L, Johnsen E, Cain C, Xu Z. Visualizing the histotripsy process: bubble cloud−cancer cell interactions in a tissue-mimicking environment. Ultrasound Med Biol 2016;42:2466–77. 27401956 10.1016/j.ultrasmedbio.2016.05.018 PMC5010997

[6] Vlaisavljevich E, Maxwell A, Warnez M, Johnsen E, Cain CA, Xu Z. Histotripsy-induced cavitation cloud initiation thresholds in tissues of different mechanical properties. IEEE Trans Ultrason Ferroelectr Freq Control 2014;61:341–52. 24474139 10.1109/TUFFC.2014.6722618 PMC4158820

[7] Xu Z, Ludomirsky A, Eun LY, Hall TL, Tran BC, Fowlkes JB, Controlled ultrasound tissue erosion. IEEE Trans Ultrason Ferroelectr Freq Control 2004;51:726–36. 15244286 10.1109/tuffc.2004.1308731 PMC2669757

[8] Xu Z, Raghavan M, Hall TL, Chang CW, Mycek MA, Fowlkes JB, High speed imaging of bubble clouds generated in pulsed ultrasound cavitational therapy-histotripsy. IEEE Trans Ultrason Ferroelectr Freq Control 2007;54:2091–101. 18019247 10.1109/TUFFC.2007.504 PMC2676886

[9] Hendricks-Wenger A, Nagai-Singer MA, Uh K, Vlaisavljevich E, Lee K, Allen IC. Employing novel porcine models of subcutaneous pancreatic cancer to evaluate oncological therapies. Methods Mol Biol 2022;2394:883–95. 35094364 10.1007/978-1-0716-1811-0_47

[10] Worlikar T, Vlaisavljevich E, Gerhardson T, Greve J, Wan S, Kuruvilla S, , editors. Histotripsy for non-invasive ablation of hepatocellular carcinoma (HCC) tumor in a subcutaneous xenograft murine model. Annu Int Conf IEEE Eng Med Biol Soc 2018;2018:6064–7. 30441719 10.1109/EMBC.2018.8513650

[11] Smolock AR, Cristescu MM, Vlaisavljevich E, Gendron-Fitzpatrick A, Green C, Cannata J, Robotically assisted sonic therapy as a noninvasive nonthermal ablation modality: proof of concept in a porcine liver model. Radiology 2018;287:485–93. 29381870 10.1148/radiol.2018171544

[12] Vlaisavljevich E, Kim Y, Owens G, Roberts W, Cain C, Xu Z. Effects of tissue mechanical properties on susceptibility to histotripsy-induced tissue damage. Phys Med Biol 2013;59:253. 24351722 10.1088/0031-9155/59/2/253 PMC4888779

[13] Knott EA, Longo KC, Vlaisavljevich E, Zhang X, Swietlik JF, Xu Z, Transcostal histotripsy ablation in an in vivo acute hepatic porcine model. Cardiovasc Intervent Radiol 2021;44:1643–50. 34244841 10.1007/s00270-021-02914-1

[14] Vlaisavljevich E, Owens G, Lundt J, Teofilovic D, Ives K, Duryea A, Non-invasive liver ablation using histotripsy: preclinical safety study in an in vivo porcine model. Ultrasound Med Biol 2017;43:1237–51. 28318889 10.1016/j.ultrasmedbio.2017.01.016

[15] Vidal-Jove J, Serres X, Vlaisavljevich E, Cannata J, Duryea A, Miller R, First-inman histotripsy of hepatic tumors: the THERESA trial, a feasibility study. Int J Hyperthermia 2022;39:1115–23. 36002243 10.1080/02656736.2022.2112309

[16] Arnold L, Hendricks-Wenger A, Coutermarsh-Ott S, Gannon J, Hay AN, Dervisis N, Histotripsy ablation of bone tumors: feasibility study in excised canine osteosarcoma tumors. Ultrasound Med Biol 2021;47:3435–46. 34462159 10.1016/j.ultrasmedbio.2021.08.004 PMC8578360

[17] Hendricks-Wenger A, Saunier S, Simon A, Grider D, Luyimbazi D, Allen IC, Histotripsy for the treatment of cholangiocarcinoma in a patient-derived xenograft mouse model. Ultrasound Med Biol 2022;48:293–303. 34750030 10.1016/j.ultrasmedbio.2021.10.002

[18] Hendricks-Wenger A, Sereno J, Gannon J, Zeher A, Brock RM, Beitel-White N, Histotripsy ablation alters the tumor microenvironment and promotes immune system activation in a subcutaneous model of pancreatic cancer. IEEE Trans Ultrason Ferroelectr Freq Control 2021;68:2987–3000. 33956631 10.1109/TUFFC.2021.3078094 PMC9295194

[19] Hendricks-Wenger A, Weber P, Simon A, Saunier S, Coutermarsh-Ott S, Grider D, Histotripsy for the treatment of cholangiocarcinoma liver tumors: in vivo feasibility and ex vivo dosimetry study. IEEE Trans Ultrason Ferroelectr Freq Control 2021;68:2953–64. 33856990 10.1109/TUFFC.2021.3073563 PMC9297335

[20] Hendricks-Wenger A, Aycock KN, Nagai-Singer MA, Coutermarsh-Ott S, Lorenzo MF, Gannon J, Establishing an immunocompromised porcine model of human cancer for novel therapy development with pancreatic adenocarcinoma and irreversible electroporation. Sci Rep 2021;11:1–14. 33828203 10.1038/s41598-021-87228-5 PMC8027815

[21] Crile GW, Hosmer HR, Rowland AF. The electrical conductivity of animal tissues under normal and pathological conditions. Am J Physiol Legacy Content 1922; 60:59–106.

[22] de Jong M, Maina T. Of mice and humans: are they the same? Implications in cancer translational research. J Nucl Med 2010;51:501–4. 20237033 10.2967/jnumed.109.065706

[23] Paigen K A miracle enough: the power of mice. Nat Med 1995;1:215–20. 7585036 10.1038/nm0395-215

[24] Rossant J, McKerlie C. Mouse-based phenogenomics for modelling human disease. Trends Mol Med 2001;7:502–7. 11689335 10.1016/s1471-4914(01)02164-5

[25] Boettcher AN, Schachtschneider KM, Schook LB, Tuggle CK. Swine models for translational oncological research: an evolving landscape and regulatory considerations. Mamm Genome 2022;33:230–40. 34476572 10.1007/s00335-021-09907-y PMC8888764

[26] Cilleros C, Dupre A, Chen Y, Vincenot J, Rivoire M, Melodelima D. Intraoperative HIFU ablation of the pancreas using a toroidal transducer in a porcine model: the first step towards a clinical treatment of locally advanced pancreatic cancer. Cancers 2021;13:6381. 34945001 10.3390/cancers13246381 PMC8699564

[27] Mauch SC, Zlevor AM, Knott EA, Couillard AB, Periyasamy S, Williams EC, Hepatic and renal histotripsy in an anticoagulated porcine model. J Vasc Interv Radiol 2023;34:386–94.e2. 36503074 10.1016/j.jvir.2022.11.034 PMC11223641

[28] Lei S, Ryu J, Wen K, Twitchell E, Bui T, Ramesh A, Increased and prolonged human norovirus infection in RAG2/IL2RG deficient gnotobiotic pigs with severe combined immunodeficiency. Sci Rep 2016;6:1–12. 27118081 10.1038/srep25222 PMC4846862

[29] Whitworth KM, Lee K, Benne JA, Beaton BP, Spate LD, Murphy SL, Use of the CRISPR/Cas9 system to produce genetically engineered pigs from in vitro-derived oocytes and embryos. Biol Reprod 2014;91:78. 25100712 10.1095/biolreprod.114.121723 PMC4435063

[30] Yugo DM, Heffron CL, Ryu J, Uh K, Subramaniam S, Matzinger SR, Infection dynamics of hepatitis E virus in wild-type and immunoglobulin heavy chain knockout JH−/− gnotobiotic piglets. J Virol 2018;92:e01208–18. 30111571 10.1128/JVI.01208-18 PMC6189505

[31] Yuan L, Jobst PM, Weiss M. Gnotobiotic pigs: from establishing facility to modeling human infectious diseases. In: Schoeb TR, Eaton KA, editors. Gnotobiotics. San Diego, CA: Academic Press; 2017. p. 349–68.

[32] Ge HY, Miao LY, Xiong LL, Yan F, Zheng CS, Wang JR, High-intensity focused ultrasound treatment of late-stage pancreatic body carcinoma: optimal tumor depth for safe ablation. Ultrasound Med Biol 2014;40:947–55. 24462161 10.1016/j.ultrasmedbio.2013.11.020

[33] Sebeke LC, Rademann P, Maul AC, Schubert-Quecke C, Annecke T, Yeo SY, Feasibility study of MR-guided pancreas ablation using high-intensity focused ultrasound in a healthy swine model. Int J Hyperthermia 2020;37:786–98. 32619373 10.1080/02656736.2020.1782999

[34] Yeats E, Gupta D, Xu Z, Hall TL. Effects of phase aberration on transabdominal focusing for a large aperture, low *f*-number histotripsy transducer. Phys Me Biol 2022;67:155004. 10.1088/1361-6560/ac7d90 PMC9396534 35772383

[35] Manresa MC, Miki H, Miller J, Okamoto K, Dobaczewska K, Herro R, A deficiency in the cytokine TNFSF14/LIGHT limits inflammation and remodeling in murine eosinophilic esophagitis. J Immunol 2022;209:2341–51. 10.4049/jimmunol.2200326 PMC10130236 36288906

[36] Li SL, Jiang P, Jiang FL, Liu Y. Recent advances in nanomaterial-based nanoplatforms for chemodynamic cancer therapy. Adv Funct Mater 2021;31:2100243.

[37] Seaman S, Zhu Z, Saha S, Zhang XM, Yang MY, Hilton MB, Eradication of tumors through simultaneous ablation of CD276/B7-H3-positive tumor cells and tumor vasculature. Cancer Cell 2017;31:501–15.e8. 28399408 10.1016/j.ccell.2017.03.005 PMC5458750

[38] Zhang C, Liu J, Guo H, Wang W, Xu M, Tan Y, Theranostic nanomedicine carrying l-menthol and near-infrared dye for multimodal imaging-guided photothermal therapy of cancer. Adv Healthc Mater 2019;8:1900409. 10.1002/adhm.201900409 31148393

[39] Vincent P, Wang H, Nieskoski M, Gunn JR, Marra K, Hoopes PJ, High-resolution ex vivo elastography to characterize tumor stromal heterogeneity in situ in pancreatic adenocarcinoma. IEEE Trans Biomed Eng 2020;67:2490–6. 31902753 10.1109/TBME.2019.2963562 PMC7494001

[40] Wang H, Mislati R, Ahmed R, Vincent P, Nwabunwanne SF, Gunn JR, Elastography can map the local inverse relationship between shear modulus and drug delivery within the pancreatic ductal adenocarcinoma microenvironmentrelationship between shear modulus and drug delivery. Clin Cancer Res 2019;25:2136–43. 30352906 10.1158/1078-0432.CCR-18-2684 PMC6445768

[41] Bardeesy N, DePinho RA. Pancreatic cancer biology and genetics. Nat Rev Cancer 2002;2:897–909. 12459728 10.1038/nrc949

[42] Hosein AN, Brekken RA, Maitra A. Pancreatic cancer stroma: an update on therapeutic targeting strategies. Nat Rev Gastroenterol Hepatol 2020;17:487–505. 32393771 10.1038/s41575-020-0300-1 PMC8284850

[43] Orsi F, Arnone P, Chen W, Zhang L. High intensity focused ultrasound ablation: a new therapeutic option for solid tumors. J Cancer Res Ther 2010;6:414. 21358073 10.4103/0973-1482.77064

[44] Zhou YF. High intensity focused ultrasound in clinical tumor ablation. World J Clin Oncol 2011;2:8. 10.5306/wjco.v2.i1.8 PMC3095464 21603311

[45] Giardino A, Innamorati G, Ugel S, Perbellini O, Girelli R, Frigerio I, Immunomodulation after radiofrequency ablation of locally advanced pancreatic cancer by monitoring the immune response in 10 patients. Pancreatology 2017;17:962–6. 29037917 10.1016/j.pan.2017.09.008

[46] Jang HJ, Lee JY, Lee DH, Kim WH, Hwang JH. Current and future clinical applications of high-intensity focused ultrasound (HIFU) for pancreatic cancer. Gut Liver 2010;4(Suppl. 1):S57. 21103296 10.5009/gnl.2010.4.S1.S57 PMC2989544

[47] Mouratidis PX, Ter Haar G. Latest advances in the use of therapeutic focused ultrasound in the treatment of pancreatic cancer. Cancers 2022;14:638. 35158903 10.3390/cancers14030638 PMC8833696

[48] Ringel-Scaia VM, Beitel-White N, Lorenzo MF, Brock RM, Huie KE, Coutermarsh-Ott S, High-frequency irreversible electroporation is an effective tumor ablation strategy that induces immunologic cell death and promotes systemic anti-tumor immunity. EBioMedicine 2019;44:112–25. 31130474 10.1016/j.ebiom.2019.05.036 PMC6606957

[49] Ruarus A, Vroomen L, Puijk R, Scheffer H, Meijerink M. Locally advanced pancreatic cancer: a review of local ablative therapies. Cancers 2018;10:16. 29320420 10.3390/cancers10010016 PMC5789366

[50] Scheffer HJ, Stam AG, Geboers B, Vroomen LG, Ruarus A, de Bruijn B, Irreversible electroporation of locally advanced pancreatic cancer transiently alleviates immune suppression and creates a window for antitumor T cell activation. Oncoimmunology 2019;8:1652532. 10.1080/2162402X.2019.1652532 PMC6791414 31646081

[51] Qu S, Worlikar T, Felsted AE, Ganguly A, Beems MV, Hubbard R, Non-thermal histotripsy tumor ablation promotes abscopal immune responses that enhance cancer immunotherapy. J Immunother Cancer 2020;8:e000200. 10.1136/jitc-2019-000200 PMC7057529 31940590

[52] Worlikar T, Zhang M, Ganguly A, Hall TL, Shi J, Zhao L, Impact of histotripsy on development of intrahepatic metastases in a rodent liver tumor model. Cancers 2022;14:1612. 35406383 10.3390/cancers14071612 PMC8996987

[53] Amrutkar M, Gladhaug IP. Pancreatic cancer chemoresistance to gemcitabine. Cancers 2017;9:157. 29144412 10.3390/cancers9110157 PMC5704175

[54] Polani F, Grierson PM, Lim KH. Stroma-targeting strategies in pancreatic cancer: past lessons, challenges and prospects. World J Gastroenterol 2021;27:2105. 34025067 10.3748/wjg.v27.i18.2105 PMC8117738

[55] Teague A, Lim KH, Wang-Gillam A. Advanced pancreatic adenocarcinoma: a review of current treatment strategies and developing therapies. Ther Adv Med Oncol 2015;7:68–84. 25755680 10.1177/1758834014564775 PMC4346211

[56] Singh MP, Sethuraman SN, Miller C, Malayer J, Ranjan A. Boiling histotripsy and insitu CD40 stimulation improve the checkpoint blockade therapy of poorly immunogenic tumors. Theranostics 2021;11:540. 33391491 10.7150/thno.49517 PMC7738858

[57] Yuan J, Ye D, Chen S, Chen H. Therapeutic ultrasound-enhanced immune checkpoint inhibitor therapy. Front Phys 2021;9:636985. 10.3389/fphy.2021.636985 PMC10664841 37994329

[58] Bailey KL, Carlson MA. Porcine models of pancreatic cancer. Front Oncol 2019;9:144. 30915276 10.3389/fonc.2019.00144 PMC6423062

[59] Bailey KL, Cartwright SB, Patel NS, Remmers N, Lazenby AJ, Hollingsworth MA, Porcine pancreatic ductal epithelial cells transformed with KRASG12D and SV40T are tumorigenic. Sci Rep 2021;11:1–13. 34183736 10.1038/s41598-021-92852-2 PMC8238942

[60] Boas FE, Nurili F, Bendet A, Cheleuitte-Nieves C, Basturk O, Askan G, Induction and characterization of pancreatic cancer in a transgenic pig model. PLoS One 2020;15:e0239391. 10.1371/journal.pone.0239391 PMC7505440 32956389

[61] Schachtschneider KM, Schwind RM, Newson J, Kinachtchouk N, Rizko M, Mendoza-Elias N, The oncopig cancer model: an innovative large animal translational oncology platform. Front Oncol 2017;7:190. 28879168 10.3389/fonc.2017.00190 PMC5572387

