# Supplementary material for: Successful In Situ Targeting of Pancreatic Tumors in a Novel Orthotopic Porcine Model Using Histotripsy
Source: Ultrasound Med Biol. Author manuscript; Available in PMC 2023 Nov 1. (PMC10529075; doi:10.1016/j.ultrasmedbio.2023.07.013)
Supplement: MMC1 [file NIHMS1921481-supplement-MMC1.pdf]

Successful *in situ* targeting of pancreatic tumors in a novel orthotopic porcine model using histotripsy

Khan Mohammad Imran[1,2], Jessica Gannon[3], Holly A. Morrison[2], Juselyn D. Tupik[2], Benjamin Tintera[2,4], Margaret A. Nagai-Singer[2], Hannah Ivester[1,2], Justin Markov Madanick[2], Alissa Hendricks-Wenger[1-3,5], Kyungjun Uh[6], David T. Luyimbazi[7], Michael Edwards[8], Sheryl Coutermarsh-Ott[2], Kristin Eden[2], Christopher Byron[9], Sherrie Clark-Deener[9], Kiho Lee[6], Eli Vlaisavljevich[3], Irving C Allen[2,*]

Supplemental data

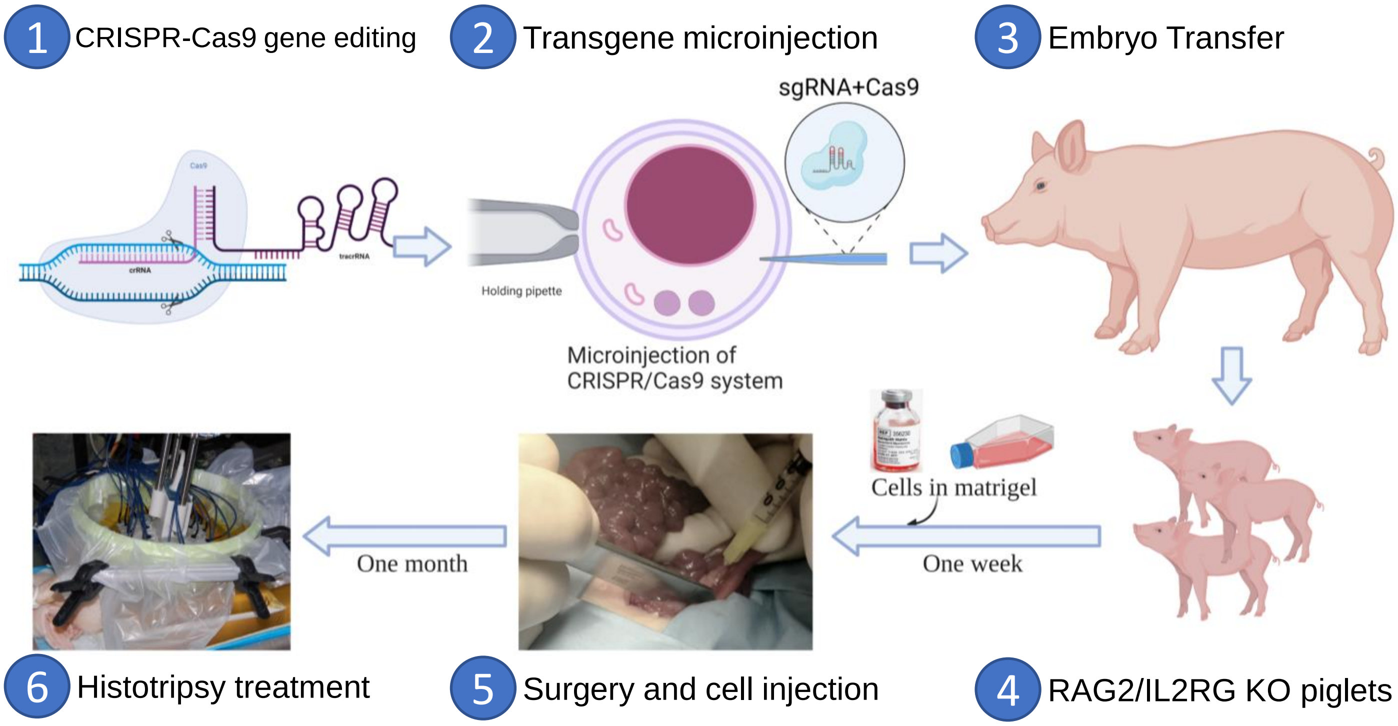

Supplemental Figure S1. Generation of immunocompromised orthotopic tumor model and treatment with histotripsy. Schematics of generation of immunocompromised pig and transplantation of human pancreatic tumor into pancreas and treatment with histotripsy.

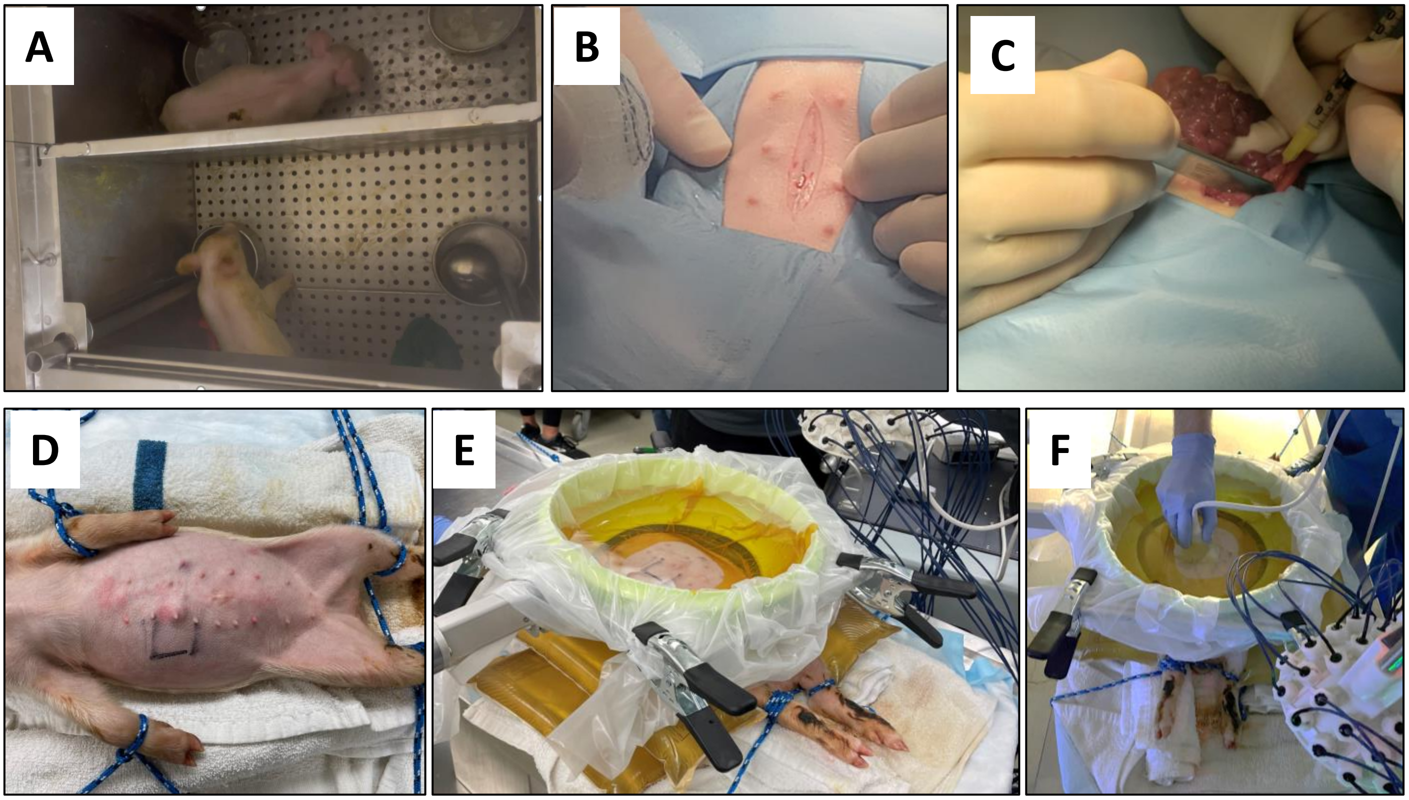

Supplemental Figure S2. Pig surgery images and histotripsy system setup. (A) Piglets in the germfree isolator (B) Piglet abdomen opening to visualize pancreas (C) Panc-1 cells being injected into the pancreas (D) Piglet area of pancreas marked after free-hand US imaging (H) Histotripsy treatment setup (I) Free-hand US imaging after histotripsy treatment.

Pig ID	Sex	RAG2 Allele 1	RAG2 Allele 2	RAG2 Allele 3	IL2RG Allele 1	IL2RG Allele 2	IL2RG Allele 3
**111**	Female	2 bp deletion	2 bp deletion		73 bp deletion, 12 bp deletion	73 bp deletion, 2 bp deletion	
**112**	Female	5 bp insertion	11 bp deletion		Not Amplified	Not Amplified	
**113**	Male	1 bp insertion (homozygous)			5 bp deletion		
**115**	Male	22 bp deletion	1 bp insertion		1 bp insertion, 34 bp deletion		
**116**	Female	7 bp deletion	2 bp deletion	1 bp insertion	2 bp deletion, 98 bp deletion	114 bp deletion	2 bp deletion
**117**	Female	2 bp deletion	1 bp insertion		9 bp deletion, 1 bp insertion		
**118**	Female	2 bp insertion (homozygous)			43 bp deletion		
**121**	Female	1 bp deletion	1 bp insertion		115 bp deletion		
**122**	Female	20 bp insertion	568 bp deletion		9 bp deletion, 3 bp deletion	1 bp insertion, 2 bp deletion	
**123**	Male	2 bp deletion (homozygous)			123 bp deletion		

Supplemental Figure S3. Pig Genotype results. The genotype of each pig was noted for the RAG2 and IL2RG allele.

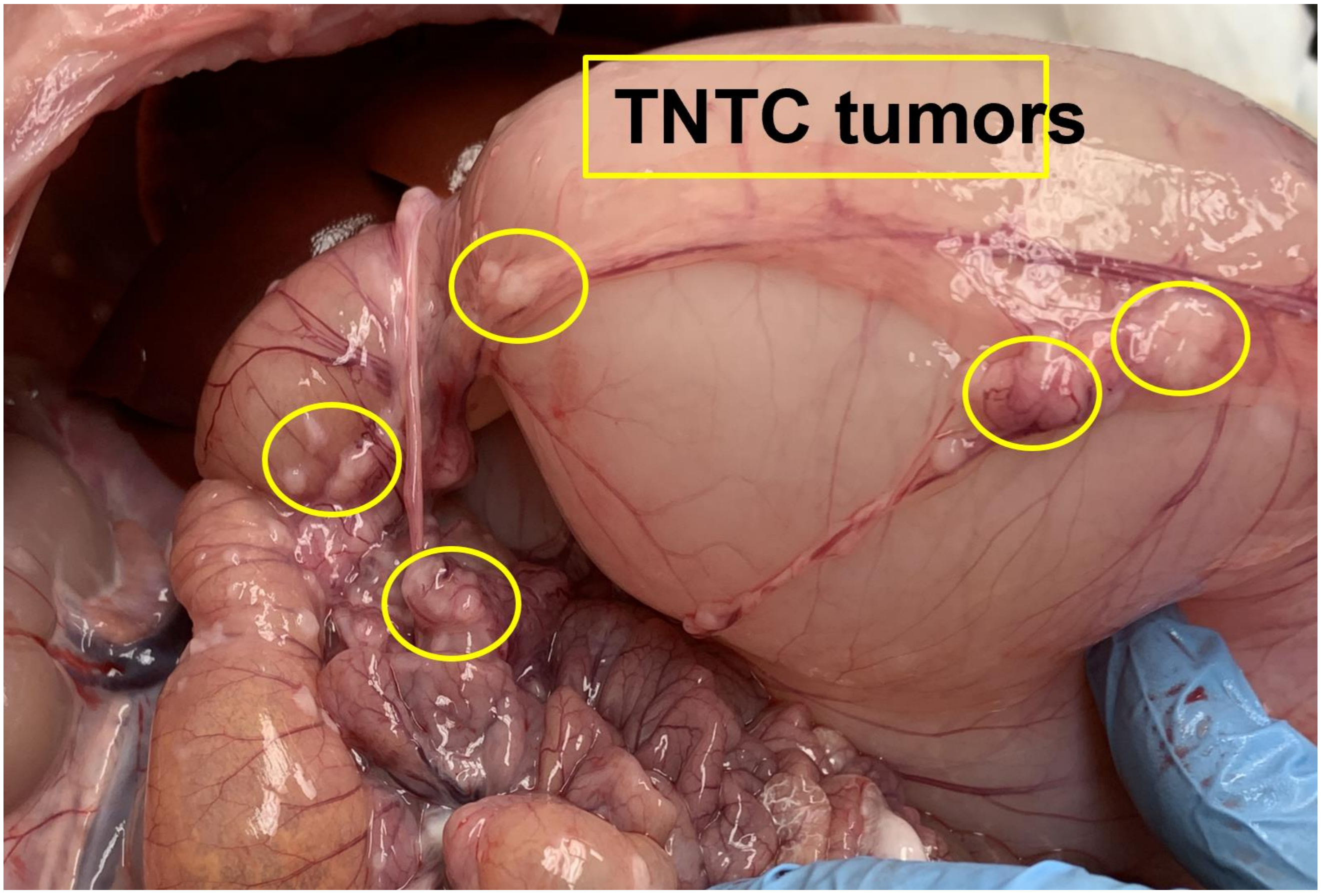

Supplemental Figure S4. Significant tumor burden and duodenal blockage was noted in one animal. During the course of the study, one animal clinically progressed more quickly than the others. Necropsy revealed significant tumor burden through the organs of the peritoneal cavity with tumors that were too numerous to count (TNTC). No tumors were found in the liver, spleen or kidney, or in any organs outside of the abdominal cavity, including the heart. The significant clinical decline in this animal was associated with a highly infiltrative tumor obstructing the duodenum causing significant gastrointestinal blockages.

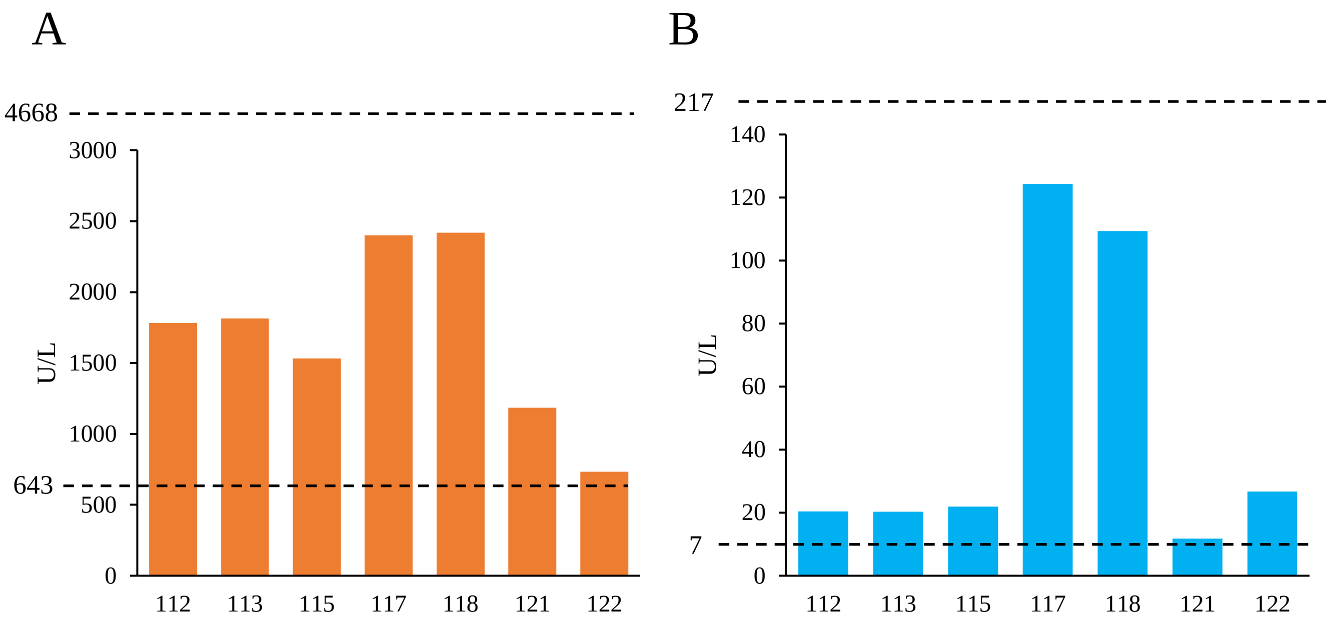

Supplemental Figure S5. Pancreatitis was not observed in any pigs. **(A)** Blood amylase levels were assessed in all animals following tumor injection. Dashed lines show the normal range of amylase in pigs (643-4668). **(B)** Blood lipase levels were assessed in all animals following tumor injection. Dashed lines show normal range of lipase in pigs (7-217).
